# Impaired T Follicular Regulatory Cell Function and Enhanced T Follicular Helper Cell Activity in Experimental Autoimmune Encephalomyelitis: Mechanistic Insights into CNS Autoimmunity

**DOI:** 10.3390/ijms27062901

**Published:** 2026-03-23

**Authors:** Gulam Hekimoglu, Kubra Sevgin, Nurullah Yucel, Gamze Yesilay, Salime Pelin Erguven, Muzaffer Seker

**Affiliations:** 1 Department of Histology and Embryology, Hamidiye International Faculty of Medicine, University of Health Sciences, Istanbul 34668, Turkey; 2 Department of Anatomy, Hamidiye Faculty of Medicine, University of Health Sciences, Istanbul 34668, Turkey; 3 Department of Molecular Biology and Genetics, Hamidiye Institute of Health Sciences, University of Health Sciences, Istanbul 34668, Turkey; 4 Turkish Academy of Sciences, Ankara 06690, Turkey

**Keywords:** EAE, CXCR5, PD-1, ICOS, FOXP3, IL-21

## Abstract

Multiple sclerosis is a chronic immune-mediated central nervous system disorder marked by neuroinflammation, demyelination, and neurodegeneration, and effectively modeled by experimental autoimmune encephalomyelitis. The objective of this study was to elucidate the roles of T follicular helper and T follicular regulatory cells in the progression of experimental autoimmune encephalomyelitis and to assess their association with IL-21 expression and central nervous system tissue pathology. In this study, experimental autoimmune encephalomyelitis was induced in 25 adult female *C57BL/6* mice. Fluorescent double immunostaining for CXCR5 in combination with PD-1, ICOS, CD4, and FOXP3 was performed, along with the analysis of IL-21 mRNA expression. Histopathological assessment was conducted on the cerebrum, cerebellum, and medulla spinalis to evaluate neuroinflammation and myelin loss. A significant increase in CXCR5^+^PD-1^+^ and CXCR5^+^ICOS^+^ T follicular helper-like cells was observed in brain tissue, indicating immune activation and T follicular helper cell involvement. Simultaneously, a marked decrease in FOXP3^+^ T follicular regulatory-like cells suggested impaired immune tolerance and enhanced autoimmune activity. The infiltration of T follicular helper-like cells was identified as a key driver of inflammation and demyelination in the central nervous system. Additionally, the elevated IL-21 mRNA expression highlighted B cell activation and the initiation of antibody-mediated responses. These findings suggest that dysregulation of the T follicular helper/T follicular regulatory axis and elevated IL-21 expression contribute to the immunopathogenesis of experimental autoimmune encephalomyelitis, providing further insight into the mechanisms underlying multiple sclerosis development.

## 1. Introduction

Multiple sclerosis is a chronic, immune-mediated central nervous system (CNS) disease characterized by neuroinflammation, demyelination, and progressive neurodegeneration, primarily affecting young adults and impacting quality of life [1]. Over 2.5 million people are affected worldwide, with incidence varying geographically. While its exact etiology remains unclear, genetic predisposition and environmental factors such as low vitamin D, smoking, and obesity contribute to susceptibility [2]. Experimental autoimmune encephalomyelitis (EAE), an animal model replicating central nervous system inflammation, demyelination, and motor deficits, has been widely used to study multiple sclerosis pathogenesis and T–B cell interactions [3]. Although T cells were initially considered the main effectors in multiple sclerosis, evidence now highlights a central role for B cells and their interactions with T cells. Autoreactive T cells have long been considered the main drivers of multiple sclerosis, causing CNS damage via inflammatory cytokines and cytotoxicity [4]. However, B cells also play a critical role by antigen-presenting, secreting cytokines, and forming intrathecal antibody aggregates that sustain CNS inflammation [5]. Therapies targeting CD19/20 B cells have shown efficacy in modulating multiple sclerosis pathology [6]. Among T cell subsets, T follicular helper (Tfh) cells are critical regulators of adaptive immunity, facilitating B cell maturation, antibody production, and germinal center formation. They express Bcl-6, CXCR5, PD-1, and ICOS and secrete cytokines, including IL-21 and IL-4 [7].

Recent studies indicate that Tfh cells contribute to multiple sclerosis and EAE pathogenesis by promoting autoreactive B cell responses. Elevated circulating Tfh cells and IL-21 levels in multiple sclerosis patients correlate with disease progression and disability [8]. In EAE, Tfh cells reside in ectopic lymphoid structures in the spinal cord, driving MOG-specific autoantibody production [8]. PD-1^+^ and ICOS^+^ Tfh cells are increased in the CNS and Cerebrospinal Fluid (CSF), correlating with disease severity [8,9]. In contrast, follicular regulatory T (Tfr) cells, which co-express CXCR5, PD-1, and FOXP3, regulate germinal center responses but are reduced and functionally impaired in multiple sclerosis [10,11]. This Tfh/Tfr imbalance contributes to dysregulated B cell activation and persistent autoimmunity. Emerging evidence indicates that Tfh cells migrate to the CNS in multiple sclerosis, contributing to local inflammation and disease progression. Upregulation of CXCL13 and its receptor, CXCR5, in the cerebrospinal fluid facilitates the recruitment and retention of Tfh cells [12]. Increased frequencies of PD-1^+^ Tfh cells in multiple sclerosis patients [9] and experimental EAE models further support their role in neuroinflammation. In EAE, PD-1^+^ and ICOS^+^ Tfh cells expand in lymphoid tissues before symptom onset and remain elevated during chronic disease [8], suggesting their involvement in both the initiation and persistence of autoimmune responses. Targeting Tfh and Tfr pathways may therefore represent a promising therapeutic avenue, motivating further investigation. Given the emerging role of Tfh cells in multiple sclerosis, targeting Tfh-related pathways offers a potential therapeutic strategy [13]. Modulation of IL-21 signaling or costimulatory molecules such as PD-1 and ICOS may limit B cell activation and autoimmunity, while enhancing Tfr cell function could help restore immune balance.

Despite these advances, the specific mechanisms by which Tfh and Tfr cells contribute to IL-21-mediated pathology in the CNS remain poorly understood. Understanding the interplay between B and T cells in multiple sclerosis is crucial for developing new therapeutic strategies. However, the interplay between Tfh and Tfr cells in the CNS and their contribution to IL-21-mediated pathology remains unclear, representing a gap in our understanding of multiple sclerosis immunopathogenesis. Therefore, this study aimed to investigate the roles of Tfh and Tfr cells, along with IL-21 expression, in the immunopathogenesis of EAE.

## 2. Results

### 2.1. Clinical Course and Disease Progression in EAE Mice

**Figure 1:**
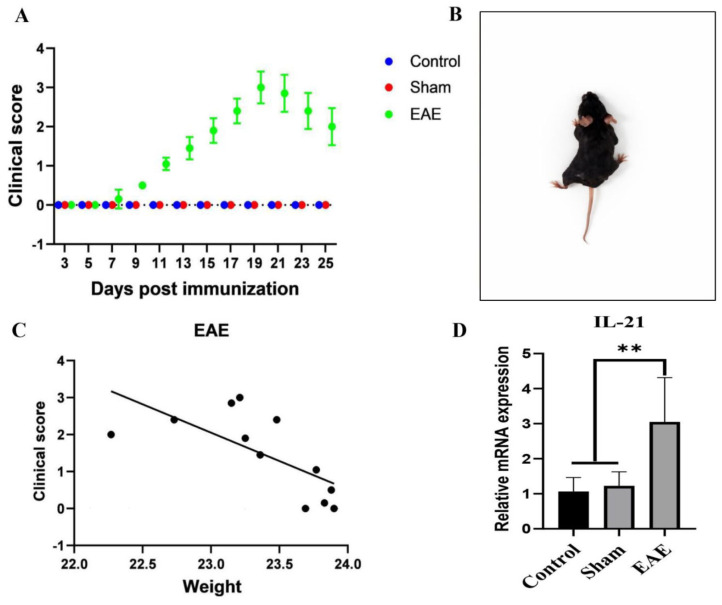
(**A**) Clinical disease course over a 25-day period following immunization. Clinical scores were analyzed using the Scheirer–Ray–Hare test. Data are expressed as mean ± standard deviation: (**B**) Representative image of a C57BL/6 mouse displaying pronounced neurological deficits after immunization. (**C**) Changes in body weight during disease progression. Mice in the EAE group showed significant weight loss that inversely correlated with increasing clinical severity, whereas the control and sham groups exhibited gradual weight gain. The relationship between weight change and clinical score was assessed using Pearson’s correlation analysis. (**D**) IL-21 mRNA expression levels in whole brain tissue, determined by RNA isolation, cDNA synthesis, and quantitative real-time PCR. Statistical analysis was performed using the Kruskal–Wallis test. IL-21 expression was significantly higher in the EAE group compared to the sham and control groups (** *p* < 0.01).

The first clinical signs of MOG_35–55_-induced EAE in *C57BL/6* mice appeared on day 11 post-immunization, and the animals were sacrificed on day 25 post-immunization. Based on the applied scoring protocol, three different phases of the disease were distinguished: onset, peak, and regression. Clinical scoring was found to be high in the EAE group. No symptoms of the disease developed in the control (*n* = 7) and sham group (*n* = 8) mice. The clinical score of the EAE group (*n* = 10) showed a significant increase day by day during the 14-day follow-up, *p* < 0.0001 (r = 0.90) (Figure 1A).

### 2.2. Clinical Severity and Weight Loss Correlate During EAE Development

After immunization, mice were monitored daily for changes in weight and clinical symptoms. The relationship between weight change and clinical score was assessed using Pearson’s correlation analysis. A significant negative correlation was observed between weight loss and EAE severity (*p* < 0.05, r = −0.69, Y = −1.534 × X + 37.34). Clinical signs appeared on day 11 post-vaccination (Video S1). In EAE C57BL/6 mice, lesions primarily affected the spinal cord, with motor symptoms progressing from the tail to rostral regions (Figure 1B). As illustrated in Figure 1C, weight loss closely reflected disease severity, confirming that motor deficits provide a reliable measure of EAE progression.

### 2.3. IL-21 mRNA Levels Increase During EAE Progression

On day 25 post-immunization, mice were anesthetized and euthanized by cervical dislocation. Brain and spinal cord tissues were harvested immediately; the right hemisphere was fixed in 10% neutral-buffered formalin for histopathological evaluation, whereas the left hemisphere was snap-frozen and stored at −80 °C for subsequent RT-PCR analyses. Following homogenization of fresh cerebrum and cerebellum tissues, total RNA was isolated. IL-21 mRNA expression levels were measured in control (*n* = 7), sham (*n* = 8), and EAE (*n* = 10) groups. The IL-21 mRNA levels (mean ± SD) were 1.06 ± 0.40 in the control group, 1.23 ± 0.40 in the sham group, and 3.05 ± 1.26 in the EAE group. IL-21 expression was significantly elevated in the EAE group compared to both the sham and control groups (Figure 1D).

### 2.4. Increased Tissue Damage in the Medulla Spinalis of EAE Mice

**Figure 2:**
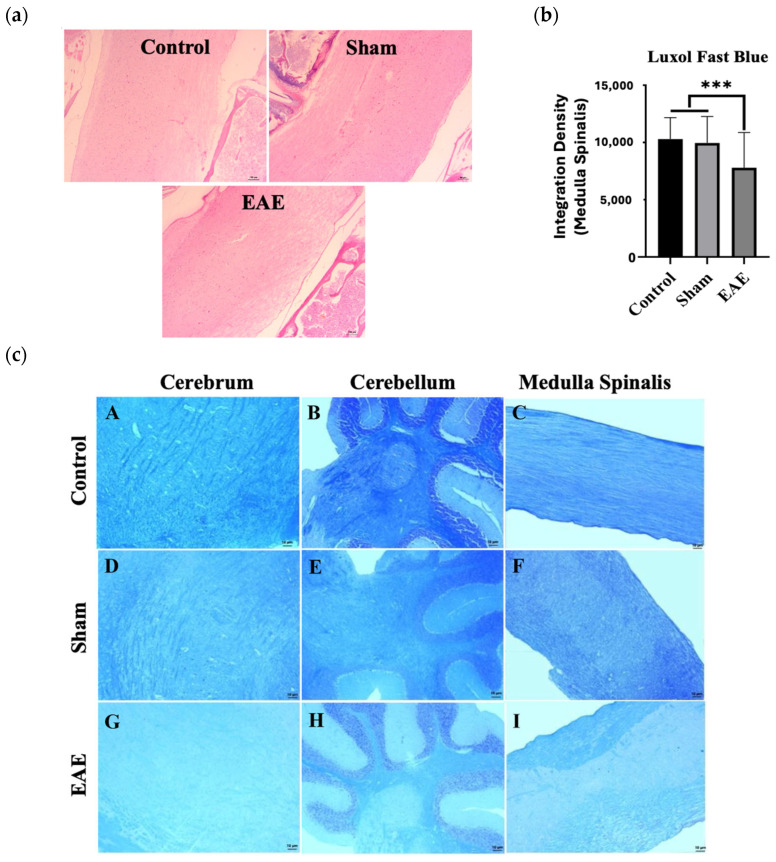
(**a**) Representative hematoxylin and eosin (H&E)-stained sections of the medulla spinalis from the control, sham, and experimental autoimmune encephalomyelitis (EAE) groups. Scale bar: 100 µm. Original magnification: 10×. (**b**) The staining intensity of Luxol Fast Blue (LFB), reflecting myelin density, was quantified using ImageJ Version 1.53a (control *n* = 7, sham *n* = 8, EAE *n* = 10; *** *p* < 0.001). (**c**) Representative LFB staining of myelin sheaths in tissue sections from the brain (**A**,**D**,**G**), cerebellum (**B**,**E**,**H**), and medulla spinalis (**C**,**F**,**I**). Myelin appeared as blue-stained regions, indicating myelin sheath integrity. Scale bar: 10 µm. Original magnification: 10×.

Hematoxylin and eosin (H&E)-stained sections revealed structural disruption and the presence of large vacuoles. Sparse lymphocyte-like cells were noted, but no obvious inflammatory cell infiltration was observed in any group, which may be characteristic of the EAE model used (Figure 2a).

### 2.5. EAE-Induced Demyelination in the Brain and Spinal Cord

Myelin loss in the spinal cord was quantified using ImageJ software (Version 1.53a: National Institutes of Health, Bethesda, MD, USA). Significant myelin loss was observed in the EAE group compared to the sham and control groups (*** *p* < 0.001, Figure 2b). Representative Luxol Fast Blue (LFB)-stained images are shown in Figure 2c.

### 2.6. Immune Cell Subset Distribution in EAE: PD-1^+^, ICOS^+^, CD4^+^, and FOXP3^+^ Cells

**Figure 3:**
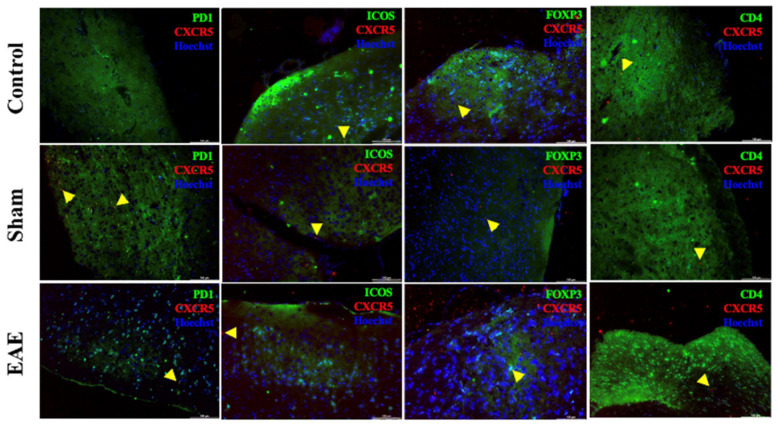
Representative sections of the cerebrum were subjected to double immunofluorescent staining using anti-CXCR5 (Alexa 594, red) in combination with PD1, ICOS, FOXP3, and CD4 (Alexa 488, green), counterstained with nuclear staining Hoechst (blue). Co-localized cells are indicated by yellow arrowheads. Scale bar: 100 µm. Original magnification: 20×.

**Figure 4:**
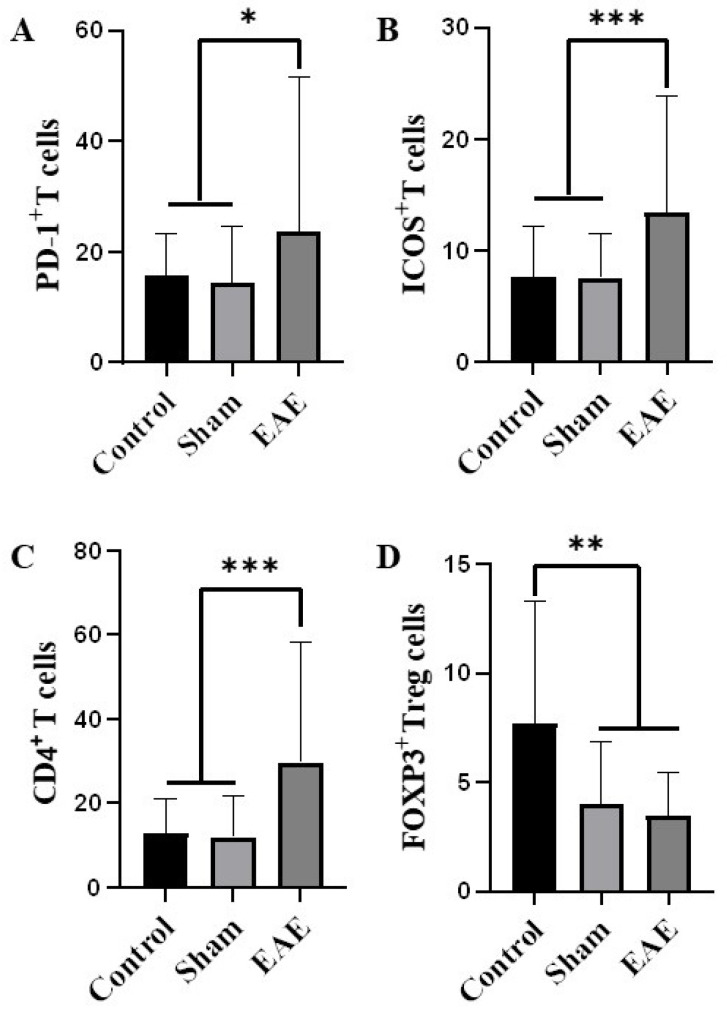
(**A**–**D**) Co-staining analyses revealed significant increases in PD-1^+^, ICOS^+^, and CD4^+^ cells, and a decrease in FOXP3^+^ cells in the cerebral cortex of the experimental autoimmune encephalomyelitis (EAE) group compared to the sham and control groups (* *p* < 0.05, ** *p* < 0.01, *** *p* < 0.001). CXCR5 expression was quite low in the entire group. Differences among groups were analyzed using the Kruskal–Wallis test with Dunn’s post hoc correction for multiple comparisons.

Immunofluorescence analysis of the cerebrum revealed significant changes in immune cell populations in EAE mice compared to the sham and control groups (Figure 3 and Figure S1). PD-1^+^ cells were markedly increased in affected regions (Figure 4A). ICOS^+^ cells were also significantly elevated (Figure 4B). CD4^+^ cells were significantly higher in EAE mice (Figure 4C). In contrast, FOXP3^+^ regulatory T (Treg)/Tfr-like cells were significantly reduced in damaged CNS regions (Figure 4D).

## 3. Discussion

Our results highlight a distinct immunological profile in EAE mice, characterized by elevated PD-1^+^, ICOS^+^, and CD4^+^ T cells and concomitant FOXP3^+^ Treg/Tfr depletion. This Tfh/Tfr imbalance correlates with clinical severity and myelin loss, providing mechanistic insight into how dysregulated adaptive immunity promotes CNS autoimmunity [14]. These observations extend previous studies on Tfh-mediated B cell activation and underscore the importance of targeting Tfh/Tfr pathways for potential therapeutic interventions in multiple sclerosis.

In the present study, tissue-resident CXCR5^+^PD-1^+^, CXCR5^+^ICOS^+^, and CXCR5^+^CD4^+^ cells were identified in the brains of EAE mice, a novel observation in our research, and their infiltration was significantly increased in the EAE mouse model. PD-1, an inhibitory immune checkpoint receptor on activated T cells, may indicate activation of self-regulatory mechanisms aimed at controlling excessive immune responses [15,16]. ICOS, a key costimulatory molecule expressed primarily on Tfh-like cells, supports Tfh activation, germinal center formation, and the regulation of antibody responses. The observed increase in CXCR5^+^ICOS^+^ Tfh-like cells suggested the initiation of B cell-mediated immunity. Importantly, this is the first study to demonstrate the presence and infiltration of CXCR5^+^PD-1^+^ and CXCR5^+^ICOS^+^ Tfh-like cells within CNS tissue in EAE, directly linking these subsets to local autoimmune pathology. These findings align with previous studies highlighting the central role of ICOS in Tfh function [17,18] and underscore the need for further investigation into the precise contributions of these cells to disease pathogenesis. Our results confirm that CXCR5^+^PD-1^+^ and CXCR5^+^ICOS^+^ Tfh-like cells are involved in EAE pathogenesis [19,20] and suggest that they may contribute to disease progression by promoting the generation of anti-MOG_35–55_ antibodies. Collectively, these Tfh-like cells and their effector molecules may serve as potential biomarkers and therapeutic targets in multiple sclerosis.

In our study, a decrease in FOXP3^+^ Tfr-like cells was observed, suggesting impaired immune tolerance and exacerbation of the autoimmune response [21]. FOXP3^+^ Tfr cells play a protective role against autoimmunity, and their reduction indicates a heightened autoimmune attack on the CNS [19]. Concurrently, we observed an increase in CXCR5^+^PD-1^+^ and CXCR5^+^ICOS^+^ Tfh-like cells, which serve as T helper cells regulating immune responses through cytokine production and interactions with other immune and CNS-resident cells. In the EAE model, CXCR5^+^PD-1^+^ and CXCR5^+^ICOS^+^ Tfh cells are key drivers of disease induction and progression [22], and their presence in the CNS reflects infiltration of activated T helper lymphocytes mediating inflammation and demyelination [23,24,25,26,27]. Additionally, IL-21 mRNA expression was elevated in the CNS, suggesting a role for IL-21 in promoting B cell differentiation and antibody production. Previous studies have shown that IL-21 synergizes to induce human plasma cell differentiation by upregulating Blimp-1 at the chromatin level [28]. While the MOG_35–55_-induced EAE model can occur in B cell-deficient mice, indicating that B cells are not required for disease initiation [29], adoptive transfer experiments suggest that B cells and Tfh cells contribute to disease progression [30]. Our findings suggest that IL-21, predominantly associated with CXCR5^+^ Tfh-like cells, may contribute to the amplification and maintenance of adaptive immune responses within the CNS during established EAE [31]. The increased IL-21 expression observed at the symptomatic stage indicates ongoing activation of proinflammatory pathways, particularly in association with CXCR5^+^ T cell responses. Although the present study does not determine whether IL-21 elevation precedes histopathological changes, the data support a role for Tfh-like cell–derived IL-21 signaling in sustaining CNS immune activation during autoimmune neuroinflammation [32].

In the preclinical stage of EAE, before significant inflammatory cell infiltration occurs in the peripheral or central nervous system, early signals of T cell activation are already detectable. Previous studies have shown that increased IL-21 and ICOS expression levels reflect early Tfh cell activation, while elevated PD-1^+^ cells suggest the initiation of immunoregulatory mechanisms [7]. These findings indicate that both proinflammatory and suppressive pathways may be simultaneously engaged prior to the onset of overt autoimmunity in EAE [33]. Molecular analyses at this early stage are valuable for understanding disease immunopathogenesis and identifying potential early therapeutic targets. In our study, the low expression of CXCR5 suggests that Tfh cell differentiation may be at an early stage. The precise role of Tfh cells in multiple sclerosis patients remains to be confirmed in future clinical studies, and additional research is needed to clarify the mechanisms driving multiple sclerosis pathogenesis and the contributions of key immune factors.

However, under certain conditions, microglia and astrocytes in the central nervous system may also express IL-21 receptors at low levels, which could contribute to local immune regulation and partially influence the observed inflammatory responses. Additionally, our analyses were largely descriptive and focused on cell numbers and cytokine expression, without directly assessing the functional interactions between Tfh cells, Tfr cells, and B cells in the CNS. The causal role of IL-21 in promoting B cell activation and antibody production was inferred but not experimentally confirmed in vivo. Moreover, while we measured early immune activation signals, the temporal dynamics and spatial localization of Tfh/Tfr interactions within germinal center-like structures in the CNS remain uncharacterized. Future studies using cell-specific markers, functional assays, and in vivo blockade or depletion models are necessary to dissect the precise mechanistic contributions of these immune subsets to EAE pathogenesis.

Several limitations of the present study should be acknowledged. First, immune cell analyses were performed at a single experimental endpoint (day 25 post-immunization), corresponding to established clinical EAE. Therefore, the temporal relationship between IL-21 expression, immune cell recruitment, and the initiation of neuroinflammation cannot be determined from our data. Longitudinal studies including earlier time points would be required to clarify the sequence of immunological events. Second, although Tfh-associated surface markers were evaluated, the absence of Bcl-6 assessment limits definitive lineage confirmation of Tfh cells in this study. While CXCR5^+^PD-1^+^/ICOS^+^ and CXCR5^+^FOXP3^+^ cell populations were identified within CNS tissue, the lineage-defining transcription factor Bcl-6 was not examined. Therefore, these populations are more appropriately interpreted as Tfh-like and Tfr-like cells rather than conclusively defined Tfh and Tfr subsets. In addition, immunofluorescence-based analysis enabled preservation of tissue architecture and visualization of in situ localization within CNS lesions; however, it provides semi-quantitative data and does not allow precise determination of subset frequencies or the Tfh/Tfr ratio, as would be achieved by multiparametric flow cytometry. Despite these limitations, the combined marker approach offers supportive phenotypic evidence for the presence of these cell populations in the inflammatory microenvironment. Future studies incorporating Bcl-6 staining and complementary multiparametric flow cytometry would further strengthen both lineage confirmation and quantitative characterization.

Despite these limitations, the present study provides in situ evidence of follicular helper- and regulatory-associated T cell phenotypes within the inflamed CNS microenvironment during established EAE.

## 4. Materials and Methods

### 4.1. Animals

Animals were obtained from Yeditepe University Faculty of Medicine Experimental Research Center and housed under standard conditions (12 h light/dark cycle, 70 cm^2^ per animal, ad libitum access to pellet feed and water). Twenty-five adult female C57BL/6 mice (19–24 g) were used, with a maximum of five mice per cage. Experimental doses were determined based on the literature [34], and the animals were divided into three groups as demonstrated in Table 1.

### 4.2. Preparation of Myelin Oligodendrocyte Glycoprotein (MOG35-55)/Complete Freund’s Adjuvant (CFA) Emulsion

Each mouse was injected with 200 μg of MOG35-55 peptide solution. Lyophilized MOG35-55 (RP10245, GenScript, Piscataway, NJ, USA) was dissolved at 2 mg/mL in double-distilled H_2_O and stored at −20 °C. CFA was diluted 1:1 and emulsified with the peptide solution by sonication (3 cycles, 70% power, 10 s) until white and viscous, then placed on ice for 30 min to ensure stability. To compensate for losses during preparation, 1.5–2 times the required volume was made. MOG_35–55_ and CFA were drawn into separate syringes (27 G for peptide, 20 G for CFA) and emulsified prior to injection [35].

### 4.3. Preparation of Pertussis Toxin

Pertussis toxin (PTx, cat no: 516560, Sigma-Aldrich, St. Louis, MO, USA) was administered intraperitoneally at 200 ng in 200 µL PBS on the day of injection and two days later. A 100 µg/mL stock solution was prepared by dissolving 50 µg PT in 500 µL double-distilled H_2_O and stored at 4 °C, then diluted 1:100 with PBS so that 100 µL contained 100 ng PT [35].

### 4.4. Animal Immunization and EAE Induction

To minimize stress and ensure optimal immunization in mice anesthetized with isoflurane, injections were performed by an experienced person. Since EAE susceptibility is affected by mouse stress, injections were performed by an experienced person in the same way to control for differences in injection technique. 200 μg of antigen/CFA emulsion was injected subcutaneously at two points on the right and left sides of the back. A bulb-shaped mass was formed under the skin, which remained throughout the experiment. 200 µL of PT was injected intraperitoneally. Mice were marked to ensure easy identification of individual mice for daily assessment. The second dose of PT was injected intraperitoneally on the second day (48 h after injection) [35].

### 4.5. Clinical Assessment

Animal weight and clinical scores were assessed daily, with weight loss serving as an early indicator of disease activity. Motor symptoms were evaluated using a 0–3 EAE scoring system by one or two blinded observers: 0, no clinical disease; 0.5, tail tip flaccidity; 1.0, flaccid tail; 1.5, flaccid tail with hindlimb inhibition; 2.0, flaccid tail with hindlimb weakness; 2.5, flaccid tail with hindlimb dragging; 3.0, flaccid tail with complete hindlimb paralysis [36].

### 4.6. Tissue Sampling and Experimental Examination of Samples

Disease onset was indicated by weight loss 1–2 days before EAE symptoms appeared. Clinical signs began on day 11 post-injection and peaked on day 19 [35]. On day 25, mice were euthanized under anesthesia by cervical dislocation, and brains and spinal cords were collected. The right hemisphere was fixed in 10% neutral-buffered formalin for histopathological evaluation, whereas the left hemisphere was snap-frozen and stored at −80 °C for subsequent RT-PCR analyses.

### 4.7. RT-PCR Analysis

Following homogenization of fresh cerebrum and cerebellum tissues, total RNA was isolated. IL-21 mRNA expression levels were measured in control (*n* = 7), sham (*n* = 8), and EAE (*n* = 10) groups. Total RNA for real-time gene expression analysis (qRT-PCR) was isolated according to the manufacturer’s instructions. RNA purity and concentration were assessed using the NanoDrop^®^ ND-100 spectrophotometer (Thermo Fisher Scientific, Wilmington, DE, USA) according to the absorbance ratios at 260 nm and 280 nm (OD260/280). Only RNA samples with purity values between 1.8 and 2.0 were considered suitable for further analysis. cDNA synthesis was performed using 500 ng of total RNA via reverse transcription. The resulting cDNA samples were diluted 1:3 in DNase- and RNase-free water before analysis. For mRNA quantification, Ct values of target genes were normalized to a control gene. IL-21-specific primers were obtained from a commercial supplier. PCR reactions were performed at a total volume of 25 μL containing 50 ng template cDNA and 2.5 μL of 10× PCR buffer. The amplification process was carried out using SYBR Green(Thermo Fisher Scientific, Wilmington, DE, USA) dye under the following conditions: an initial denaturation step at 95 °C for 5 min, followed by 40 cycles of denaturation at 95 °C for 5 s and annealing/extension at 60 °C for 10 s. GAPDH gene expression was used as an internal control. The specificity of amplification was confirmed by melting curve analysis. Gene expression levels were determined using the comparative threshold (Ct) method, specifically the ΔΔCt method, and relative expression changes were calculated using the 2^(−ΔΔCt)^ equation. Each qRT-PCR experiment was performed in triplicate, and the data are presented as the mean ± standard deviation (SD).

### 4.8. Histological and Immunofluorescence Staining

#### 4.8.1. Routine Histology

For histopathological evaluation, tissues were paraffin-processed tissue blocks were sectioned at 4 µm and dehydrated through an ascending alcohol series (70–100%). For general morphology, sections were stained with hematoxylin and eosin, cleared in xylene, and mounted with Entellan. For myelin/myelinated axon visualization, sections were stained with LFB at 56 °C overnight, rinsed with 95% ethanol, differentiated in lithium carbonate and 70% ethanol, and counterstained with Cresyl Violet. After dehydration and clearing, sections were mounted with Entellan and examined under a light microscope. 5 images of the cerebrum, 3 images of the cerebellum, and 3 images of the medulla spinalis white matter were taken from LFB staining sections. Myelin loss was evaluated with ImageJ software. Integrated density was selected as the quantitative metric for LFB staining because it reflects both the stained area and staining intensity, thereby providing a more comprehensive assessment of myelin content.

#### 4.8.2. Immunofluorescence Staining

Paraffin-embedded biopsy samples were sectioned into 4 µm thick slices. Following deparaffinization, antigen retrieval was performed by boiling the tissue samples in a 10% citrate-buffered solution under pressure. The samples were incubated with the following primary antibodies at 4 °C: ant-CD4 (1:200) (A0362, Abclonal, Wuhan, China), anti-FOXP3 (1:200) (A1205, Abclonal, Wuhan, China), anti-ICOS (1:200) (A1811, Abclonal, Wuhan, China), anti-PD-1 (1:200) (A11973, Abclonal, Wuhan, China), and anti-CXCR5 (1:200) (sc-37375, Santa Cruz Biotechnology, Dalla, TX, USA). After primary antibody incubation, the samples were biotinylated, followed by secondary antibody incubation. We used Alexa Fluor 488 conjugated secondary antibody (1:200, Thermo Fisher) for secondary antibodies and green fluorescence and Alexa Fluor 594 conjugated secondary antibody (1:200, Thermo Fisher) for red fluorescence. For nucleus staining, we used Hoechst 33342 Solution (1:1000) (cat no: 62249, Thermo Scientific) for five minutes. For negative controls, the same protocol was followed except that primary antibodies were omitted. Images were acquired using a Leica DM8i S immunofluorescence microscope (Leica Microsystems, Wetzlar, Germany) equipped with appropriate fluorescence filter sets. Quantification of immunofluorescence-positive cells and colocalization analysis were performed using a custom-developed analysis tool under blinded conditions. Fluorescence intensity thresholds were kept constant for all sections analyzed. Five of the cerebrum and cerebellum images from each mouse were evaluated. The number of CXCR5^+^, PD-1^+^, ICOS^+^, CD4^+^, and FOXP3^+^ cells was counted.

### 4.9. Statistical Analysis

The Scheirer–Ray–Hare test was used for the clinical score. Real-time PCR was analyzed by the Kruskal–Wallis test. A Pearson correlation test was performed between weight change and the severity of clinical symptoms. All values are expressed as median (IQR). All analyses were performed using GraphPad Prism 8.4.2 software (GraphPad, San Diego, CA, USA). *p*-values < 0.01 were considered statistically significant.

## 5. Conclusions

This study demonstrates that both proinflammatory and immunoregulatory mechanisms are activated early in the EAE model, before the onset of clinical symptoms. Elevated IL-21 and ICOS levels indicate early Tfh cell activation, while increased PD-1^+^ cells reflect the initiation of immune regulatory responses. The observed decrease in FOXP3^+^ Tfr-like cells alongside increased CXCR5^+^PD-1^+^ and CXCR5^+^ICOS^+^ Tfh-like cells suggests a shift toward heightened autoimmune activity. These findings highlight the critical role of Tfh cells and IL-21 in EAE pathogenesis and support their potential as biomarkers and therapeutic targets in multiple sclerosis. Overall, our results provide evidence that T cell activation, immune regulation, and B cell priming occur at early stages of disease, offering valuable insights for the development of novel diagnostic and treatment strategies for multiple sclerosis.

## Figures and Tables

**Table 1:** Experimental groups and treatment protocols used in the study.

Group	Condition	*n*	Treatment	Route and Dose
1	Control	7	PBS	Subcutaneous (Right 100 μL + Left 100 μL)
				Intraperitoneal (100 μL + 48 h later 100 μL)
2	Sham	8	CFA	Subcutaneous (Right 100 μL + Left 100 μL)
			PTX	Intraperitoneal (100 μL+ 48 h later 100 μL)
3	EAE	10	CFA + MOG	Subcutaneous (Right 100 μg/100 μL + Left 100 μg/100 μL)
			PTX	Intraperitoneal (100 μL + 48 h later 100 μL)

## Data Availability

The datasets generated and/or analyzed during the current study are available from the corresponding author upon reasonable request.

## Supplementary Materials

The following supporting information can be downloaded at https://www.mdpi.com/article/10.3390/ijms27062901/s1.
