# Supplementary figures and images for: Impaired T Follicular Regulatory Cell Function and Enhanced T Follicular Helper Cell Activity in Experimental Autoimmune Encephalomyelitis: Mechanistic Insights into CNS Autoimmunity
Source: Int J Mol Sci. 2026 Mar 23;27(6):2901. doi: 10.3390/ijms27062901 (PMC13027149; doi:10.3390/ijms27062901)

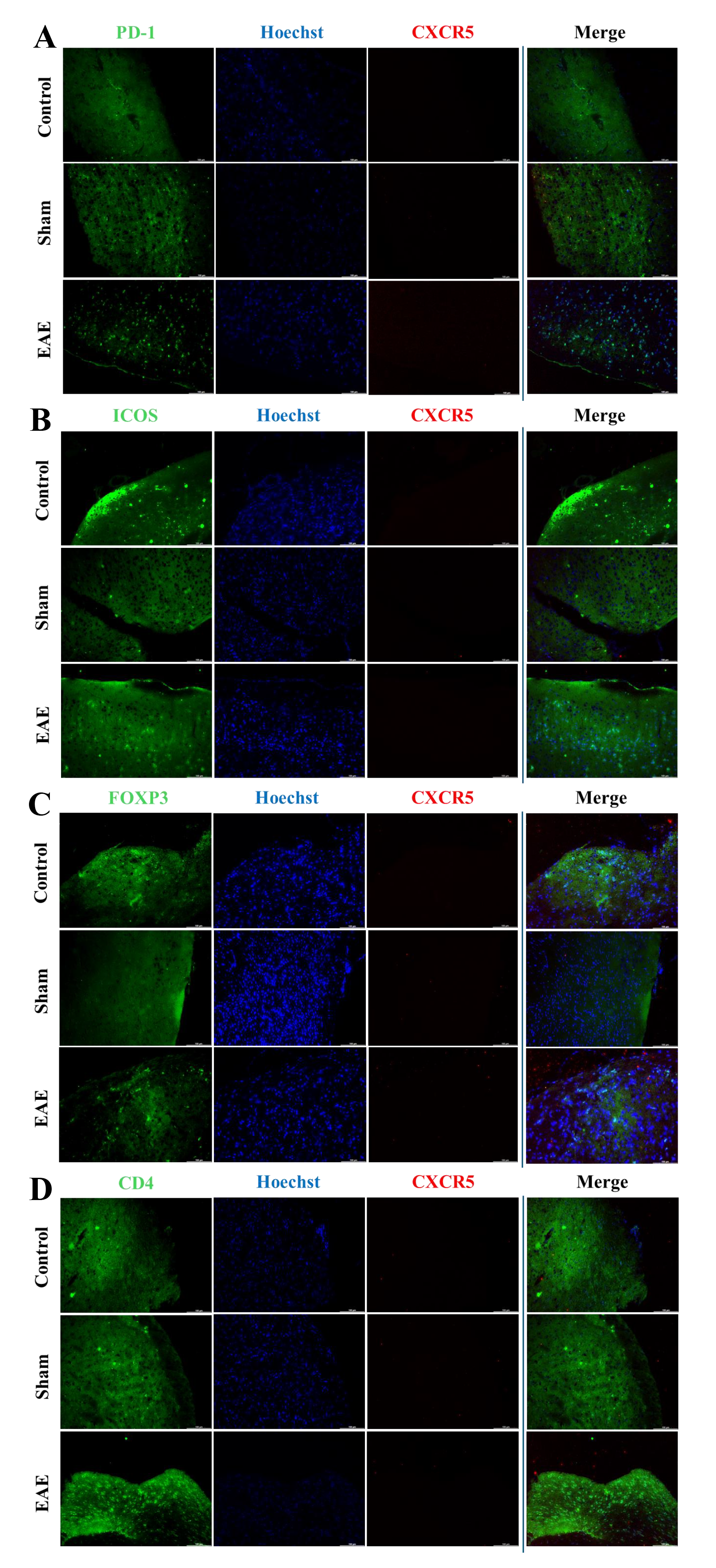

Supplement: Supplementary file 1 [file ijms-27-02901-s001.zip › Figure S1.tif]
